# *PaCYP78A9*, a Cytochrome P450, Regulates Fruit Size in Sweet Cherry (*Prunus avium* L.)

**DOI:** 10.3389/fpls.2017.02076

**Published:** 2017-12-05

**Authors:** Xiliang Qi, Congli Liu, Lulu Song, Yuhong Li, Ming Li

**Affiliations:** Zhengzhou Fruit Research Institute, Chinese Academy of Agricultural Sciences, Zhengzhou, China

**Keywords:** *Prunus avium* L, CYP78A, fruit size, VIGS, *PaCYP78A9*

## Abstract

Sweet cherry (*Prunus avium* L.) is an important fruit crop in which fruit size is strongly associated with commercial value; few genes associated with fruit size have, however, been identified in sweet cherry. Members of the CYP78A subfamily, a group of important cytochrome P450s, have been found to be involved in controlling seed size and development in *Arabidopsis thaliana*, rice, soybean, and tomato. However, the influence of CYP78A members in controlling organ size and the underlying molecular mechanisms in sweet cherry and other fruit trees remains unclear. Here, we characterized a *P. avium* CYP78A gene *PaCYP78A9* that is thought to be involved in the regulation of fruit size and organ development using overexpression and silencing approaches. *PaCYP78A9* was significantly expressed in the flowers and fruit of sweet cherry. RNAi silencing of *PaCYP78A9* produced small cherry fruits and *PaCYP78A9* was found to affect fruit size by mediating mesocarp cell proliferation and expansion during fruit growth and development. Overexpression of *PaCYP78A9* in *Arabidopsis* resulted in increased silique and seed size and *PaCYP78A9* was found to be highly expressed in the inflorescences and siliques of transgenic plants. Genes related to cell cycling and proliferation were downregulated in fruit from sweet cherry *TRV::PaCYP78A9*-silencing lines, suggesting that *PaCYP78A9* is likely to be an important upstream regulator of cell cycle processes. Together, our findings indicate that *PaCYP78A9* plays an essential role in the regulation of cherry fruit size and provide insights into the molecular basis of the mechanisms regulating traits such as fruit size in *P. avium*.

## Introduction

Sweet cherry (*Prunus avium* L.) is an economically valuable horticultural crop that is widely cultivated in temperate regions; its fleshy fruits are recognized as having nutraceutical properties and antioxidant activity (Li et al., 2010). As large fruit size in sweet cherry generates a premium market price (Whiting et al., 2006), increasing cherry fruit size has long been one of the most important goals for breeding selection during domestication and modern horticultural crop breeding (Zhang et al., 2010). Additional insight into the genetic and molecular mechanisms responsible for controlling sweet cherry fruit should, therefore, help to inform strategies to acquire larger fruit. While several previous studies have determined that fruit size is controlled by multiple genetic loci in sweet cherry and other horticultural fruit trees (Zhang et al., 2010; Rosyara et al., 2013; Campoy et al., 2015), only a few genes related to the molecular mechanisms regulating fruit size have thus far been identified. Thus, characterization of genes associated with fruit size and the molecular mechanisms that determine final fruit size is urgently required to assist in the development of strategies to increase fruit size.

Plant organ size is one of the most important agronomical traits targeted during domestication. Plant organ growth and development are genetically determined by both cell division and cell expansion (Horiguchi et al., 2006; Li and Li, 2015, 2016; Si et al., 2016). Several studies have suggested that organ size, including seed and fruit size, is ultimately controlled by multiple factors such as plant hormones, ubiquitin, microRNAs, and cytochrome P450s (CYPs) (Fang et al., 2012; Nitsch et al., 2012; Du et al., 2014; Ma et al., 2015, 2016; Yao et al., 2015). For instance, AUXIN RESPONSE FACTOR 2 (ARF2) and DA1 (DA means ‘large’ in Chinese, ubiquitin receptor) limit cell proliferation in the integuments of ovules and developing seeds, ultimately maternally affecting seed size (Schruff et al., 2006; Li et al., 2008). Abscisic acid (ABA)-biosynthesis related ABSCISIC ACID DEFICIENT2 (ABA2) and ABSCISIC ACID-INSENSITIVE5 (ABI5) regulate seed size by mediating embryonic cell proliferation and early endosperm cellularization in early seed development (Cheng et al., 2014). TRANSPARENT TESTA GLABRA2 (TTG2) and APETALA2 (AP2) play important roles in controlling seed size and growth by influencing cell elongation in the maternal integuments (Johnson, 2002; Jofuku et al., 2005). The RING-type E3 ubiquitin ligases, ENHANCER OF DA1 (EOD1) and DA2 regulate seed size by restricting cell proliferation in the maternal integuments (Li et al., 2008; Xia et al., 2013). MicroRNA172 (miRNA172) governs floral organ development and organ size by inhibiting translation of *APETALA2* (*AP2*) (Yao et al., 2015).

Cytochrome P450s is one of the largest enzymatic protein families that is found in land plants from bryophytes to angiosperms; members of this family play vital roles in a variety of metabolic pathways by producing primary and secondary metabolites such as sterols, isoflavonoids, terpenoids, flavonoids, steroids, anthocyanins, phytohormones, and phytoalexin to promote plant growth and development and protect plants from stress (Ohkawa et al., 1998; Li et al., 2012; Xu J. et al., 2015). CYP78A, an important CYP subfamily, is a highly conserved plant-specific gene family (Nelson, 2006; Mizutani and Ohta, 2010). In *Arabidopsis thaliana*, six *CYP78A* genes, *CYP78A5*, *CYP78A6*, *CYP78A7*, *CYP78A8*, *CYP78A9*, and *CYP78A10*, have been found to be involved in the control of organ growth and development (Wang et al., 2008; Adamski et al., 2009; Bak et al., 2011; Fang et al., 2012; Sotelo-Silveira et al., 2013); several CYP78As have also been found to regulate organ size and development in other plants such as rice, wheat, tomato, and soybean (Chakrabarti et al., 2013; Nagasawa et al., 2013; Ma et al., 2015, 2016; Xu F. et al., 2015; Zhao et al., 2016). For example, KLUH/CYP78A5 regulated seed or fruit size by promoting integument cell proliferation in *Arabidopsis*, tomato, wheat, and soybean (Adamski et al., 2009; Chakrabarti et al., 2013; Ma et al., 2016; Zhao et al., 2016); TaCYP78A3 controls seed size by increasing cell proliferation in the integument in wheat (Ma et al., 2015); and OsCYP78A13 regulates grain size by mediating cell-cycle progression to balance the embryo/endosperm size in rice (Nagasawa et al., 2013). AtCYP78A9 acting redundantly with EOD3/CYP78A6 and CYP78A8 plays a critical role in regulating floral organ growth and integument development by promoting cell proliferation, thereby ultimately affecting seed size in *A. thaliana* (Fang et al., 2012; Sotelo-Silveira et al., 2013). However, the role of CYP78A members in the control of organ size and development has not yet been reported in sweet cherry or other fruit trees.

In this study, the role of *P. avium* PaCYP78A9, a CYP78A member, in the regulation of fruit size and organ development in sweet cherry was characterized using overexpression and silencing approaches. Fruit expression levels of *PaCYP78A9* were detected in a landrace sweet cherry, ‘Longguan,’ and a wild sweet cherry, ‘Mazzard,’ respectively. *PaCYP78A9* overexpression in *Arabidopsis* accelerated silique and seed development, resulting in enlarged seeds. Moreover, silencing of *PaCYP78A9* during sweet cherry fruit development caused decreases in fruit mesocarp cell number and size, leading to a reduction in fruit size (Kawai et al., 2016; Cui and Wang, 2017). Together, our results provide direct evidence that *PaCYP78A9* is involved in the regulation of fruit size; these results further contribute to an understanding of the cellular basis and genetic regulation of sweet cherry fruit size and development that may assist in the generation of new lines in the future with increased yield.

## Materials and Methods

### Plant Materials

Two *P. avium* varieties, a wild sweet cherry, ‘Mazzard’, and a landrace sweet cherry, ‘Longguan’, were grown in the resource garden of the National Fruit Tree Germplasm Repository, Zhengzhou Fruit Research Institute, Chinese Academy of Agricultural Sciences (Zhengzhou, China). A single fruit of ‘Longguan’, a cultivated larger cherry, weighs more than 8 g, while ‘Mazzard’, a wild forest cherry of northern European origin, possesses very small fruit (∼2 g). The *Arabidopsis cyp78a9* insertional mutant used in this study was provided by Prof. Huixian Zhao and Dr. Meng Ma from the College of Life Sciences, Northwest A (Agriculture) & F (Forestry) University in Yangling Shaanxi, China. *A. thaliana* used in this study was preserved in our laboratory. All *Arabidopsis* plants were grown at 20–22°C in a greenhouse with a 16 h light/8 h dark cycle and 60–75% relative humidity.

### *Pacyp78a9* Gene Isolation and Phylogenetic Analysis

Total RNA was extracted and purified from the leaves and fruit of sweet cherry using an EASYspin RNA Plant RNA rapid extraction kit (Yuanpinghao Bio, Tianjin, China) according to the manufacturer’s protocol. First-strand cDNA was synthesized from purified RNA samples using the FastQuant RT (With gDNase) kit (Tiangen Bio, Beijing, China) in accordance with the manufacturer’s instructions. Specific primers (*PaCYP78A9-F* and *PaCYP78A9-R*) were used to amplify the full-length *PaCYP78A9* gene from the first-strand cDNA. MEGA 5.01 software was used to construct the phylogenetic tree of the PaCYP78A9 protein, other reported CYP78A family proteins from *Triticum urartu*, *A. thaliana*, *Oryza sativa*, and unreported CYP78A family proteins from the completed genome-sequencing fruit tree, including peach, apple, pear, grape; this was done using the neighbor-joining method with 500 bootstrap replicates (Tamura et al., 2011).

### Vector Construction

The pBI121-*PaCYP78A9* expression vector *p35S::PaCYP78A9* was constructed by In-Fusion cloning as described previously (Frandsen et al., 2012). Full-length *PaCYP78A9* was amplified using the primer pairs *PaCYP78A9-e-F* and *PaCYP78A9-e-R* that included 16-bp-long 5′ overhangs identical to the corresponding pBI121 sequence digested with *Xba*I or *BamH*I. The vector pBI121 was digested with *Xba*I and *BamH*I. Next, the full-length *PaCYP78A9* fragment and the digested pBI121 were fused together with the In-Fusion^TM^ HD Cloning kit (Clontech, Mountain View, CA, United States) to obtain the *p35S::PaCYP78A9* plasmid.

A 469-bp fragment of *PaCYP78A9* was amplified (bases 511–980) with the primers *PaCYP78A9-RNAi-F* and *PaCYP78A9-RNAi-R* containing the *Eco*RI and *Kpn*I restriction enzyme sites, respectively. The *PaCYP78A9* fragment was inserted into a *TRV2* vector digested with *Xba*I and *BamH*I using T4 ligase to construct the *TRV:PaCYP78A9* plasmid.

### *Arabidopsis* Transformation

The expression vector *p35S::PaCYP78A9* was introduced into the *Arabidopsis* Columbia ecotype or the *Arabidopsis cyp78a9* insertional mutant by the *Agrobacterium tumefaciens*-mediated floral-dip method when the *Arabidopsis* plants were 30-days-old. The seeds of transformants were collected and screened on MS medium supplemented with 50 mg/L kanamycin. Kanamycin-resistant plants were selected and transferred to growth soil chambers in a greenhouse at 20–22°C with a 16 h light/8 h dark cycle and 60–75% relative humidity where they were grown until maturity. The T3 generation of transgenic *PaCYP78A9* overexpression and *cyp78a9*-complementation lines were used for further phenotypic analysis.

### TRV-Mediated *Pacyp78a9* Gene Silencing in Cherry Fruit

TRV-mediated *PaCYP78A9* gene silencing was performed as described previously with *Agrobacterium tumefaciens-*mediated transformation with the following modifications for cherry fruit (Fu et al., 2005; Shen et al., 2014; Li et al., 2015). (i) *Agrobacterium* strain GV3101 were cultured overnight at 28°C to OD_600_ of 0.8, and were resuspended in the *Agrobacterium* infiltration buffer (10 mM MgCl_2_, 10 mM MES, pH 5.6, 100 μM acetosyringone) to a final OD_600_ of 0.8–1.0. (ii) Mixed *Agrobacterium* GV3101 strains containing the *pTRV1*, *pTRV2*, or *pTRV:PaCYP78A9* vectors were infiltrated with a needle-less syringe into the basal pedicel of cherry fruit 7 days after full bloom (DAFB) until the whole fruit was permeated. (iii) The inoculated fruits were treated with bagging for 2 days. Fruits were evaluated at 10 days post-inoculation (dpi). The experiment was repeated three times with 30 transformed fruits per strain per replicate come from the same tree of 10 years old.

### Semi-Quantitative Reverse Transcription PCR (RT-PCR) and Quantitative Real-Time PCR (qRT-PCR) Analysis

Reverse transcription PCR and qRT-PCR assays were performed as previously described by Qi et al. (2015). Total RNA from different plant tissues (leaf, flower-bud, blossom, and fruit) were isolated as described above. A FastQuant RT (With gDNase) kit (Tiangen Bio) was used to synthesize the cDNA from RNA. The expression levels of target genes were quantified relative to the constitutively expressed *Histone2* (Pav_sc0000671.1) gene using qRT-PCR in an ABI7500 PCR thermocycler (Applied Biosystems, Foster City, CA, United States) with the TransStart Top Green qPCR SuperMix (TransGen Biotech, Beijing, Chain) containing SYBR Green fluorescent intercalating dye.

Equal amounts of RNA from sweet cherry fruit harvested from the *TRV::PaCYP78A9-* and *TRV::00*-silencing lines were used for reverse transcription of the first-strand cDNA via a FastQuant RT (With gDNase) kit (Tiangen Bio) according to the manufacturer’s protocol. RT-PCR was performed with equal amounts of cDNA as previously described, and using *Histone2* as an internal control. The primers used in the RT-PCR and qRT-PCR assays are listed in Supplementary Table S1. The expression level was calculated from three replicates.

### Morphological and Cytological Characterization

Morphological analysis of plant organs, including organ size and organ weight, was conducted according to the methods described previously (Disch et al., 2006; Adamski et al., 2009; Chakrabarti et al., 2013).

To assess cytologically the fruit cells from the exocarp and endocarp of sweet cherry fruit, morphological and cytological observations were performed as previously described with some modifications using pericarp tissue samples taken from sweet cherry fruit harvested from *TRV::PaCYP78A9-* and *TRV::00*-silencing lines at 40 DAFB (Adamski et al., 2009; Chakrabarti et al., 2013; Rojas-Gracia et al., 2017). Whole fresh cherry fruit were fixed in FAA solution (5% formaldehyde, 5% acetic acid, and 50% ethanol) at 4°C overnight after vacuuming. The samples were then dehydrated through a graded ethanol concentration series composed of 75% ethanol for 4 h, 85% ethanol for 2 h, 90% ethanol for 2 h, 95% ethanol for 1 h, and finally absolute ethanol for 1 h before being transitioned into histo-clear xylene for 20 min. The fresh tissue samples were embedded in paraffin using TKY-BMB (JB-T5, Wuhan Junjie Equipment Factory, Wuhan, China), and sectioned (4 μm in thickness) using a rotary microtome (Leica RM2016; Leica Microsystems, Germany). Subsequently, the paraffin sections were dewaxed with xylene and stained with hematoxylin and eosin, followed gradient alcohol dehydration, vitrification by xylene, and neutral gum sealing. Finally, the samples of stained cherry fruit epicarp and mesocarp sections were observed using light microscopy (Microscope Nikon Eclipse Ci, Nikon, Tokyo, Japan) with a Nikon DS-U3 digital camera (Nikon, Tokyo, Japan). Three replicates were performed for each sample.

### Statistical Analysis

All data of results are provided as the mean ± standard deviation (SD). Statistically significant differences indicated in figures were based on *t*-test or one-way ANOVA (*p* ≤ 0.05) using SPSS 17.0 version (SPSS Inc., Chicago, IL, United States). Figures were made by Origin 9.1 (Microcal Software Inc., Northampton, MA, United States).

## Results

### Phylogenetic Analysis of CYP78A in Plants

**FIGURE 1:**
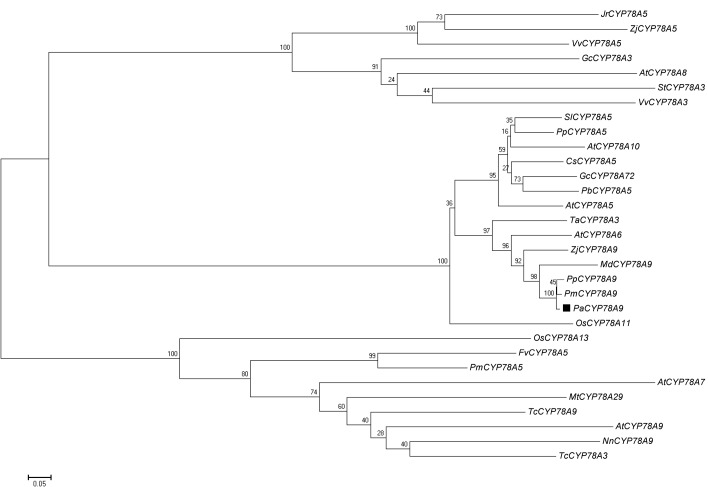
Phylogenetic analysis of the *Prunus avium* PaCYP78A protein and CYP78A orthologs in other plant species. Source organisms and accession numbers for homologous CYP78A proteins were as follows: JrCYP78A5 (*Juglans regia*, XM_018969352.1), ZjCYP78a5 (*Ziziphus jujuba*, XM_016027740.1), VvCYP78A5 (*Vitis vinifera*, XM_002265274.3), GcCYP78A3 (*Glycine max*, NM_001317595.1), AtCYP78A8 (*Arabidopsis thaliana*, NM_100001.2), StCYP78A3 (*Solanum tuberosum*, XM_006339692.2), VvCYP78A3 (*V. vinifera*, XM_002266457.2), SlCYP78A5 (*S. lycopersicum*, XM_004236016.3), PpCYP78A5 (*Prunus persica*, XM_007209835.2), AtCYP78A10 (*A. thaliana*, NM_106071.1), CsCYP78A5 (*Citrus sinensis*, XM_006477669.1), GcCYP78A72 (*Glycine max*, XM_003554632.3), PbCYP78A5 (*Pyrus* × *bretschneideri*, XM_009358148.2), AtCYP78A5 (*A. thaliana*, NM_101240.4), TaCYP78A3 (*Triticum aestivum*, KP768392.1), AtCYP78A6 (*A. thaliana*, NM_130231.4), ZjCYP78A9 (*Ziziphus jujuba*, XM_016033300.1), MdCYP78A9 (*Malus* × *domestica*, XM_008345221.2), PpCYP78A9 (*P. persica*, XM_020556145.1), PmCYP78A9 (*P. mume*, XM_008234862.2), PaCYP78A9 (*P. avium*, XM_021959332.1), OsCYP78A11 (*Oryza sativa*, XM_015757991.1), OSCYP78A13 (*O. sativa*, XM_015790749.1), FvCYP78A5 (*Fragaria vesca*, XM_004298317.2), PmCYP78A5 (*P. mume*, XM_008241890.2), AtCYP78A7 (*A. thaliana*, NM_121034.2), Mt CYP78A29 (*Medicago truncatula*, DQ335794.1), TcCYP78A9 (*Theobroma cacao*, XM_007051407.2), AtCYP78A9 (*A. thaliana*, NM_116053.3), NnCYP78A9 (*Nelumbo nucifera*, XM_010269146.1), TcCYP78A3 (*T. cacao*, XM_018117954.1). The tree was constructed using MEGA (version 5.01). A neighbor-joining evolutionary phylogeny test and 500 bootstrap replicates were selected for the analysis. The scale bar represents 0.2 substitutions per site.

To understand the cellular and molecular mechanism underlying fruit size in sweet cherry (*P. avium*), the peach genome v.2.1 sequence released by the International Peach Genome Initiative (GDR database^1^) was used to isolate the *PaCYP78A9* gene from *P. avium*. An open reading frame 1653 bp was obtained for *PaCYP78A9* with a start codon ATG and a stop codon TAA; this encoded a protein with 550 amino acid residues. To better understand the relationships between PaCYP78A9 and other members of the CYP78A family, multiple sequence alignment of the PaCYP78A9 protein and other CYP78A family members from various plant species was used to construct a phylogenetic tree. This showed that PaCYP78A9 was closely related to PmCYP78A9, PpCYP78A9, and MdCYP78A9, which have not been functionally characterized, and displayed high similarity to *Arabidopsis* CYP78A9 (67% identity), CYP78A6 (66% identity), CYP78A8 (65% identity), and to wheat CYP78A3 (59% identity), to tomato CYP78A5 (52% identity, **Figure 1**). These results demonstrated that PaCYP78A9 in *P. avium* is an ortholog of PpCYP78A9, PmCYP78A9, and MdCYP78A9, and might have the same function as AtCYP78A9 (Sotelo-Silveira et al., 2013) and TaCYP78A3 (Ma et al., 2015) that are known to play critical roles in regulating seed size.

### Expression Patterns of *PaCYP78A9* during Fruit Growth and Development in Sweet Cherry

**FIGURE 2:**
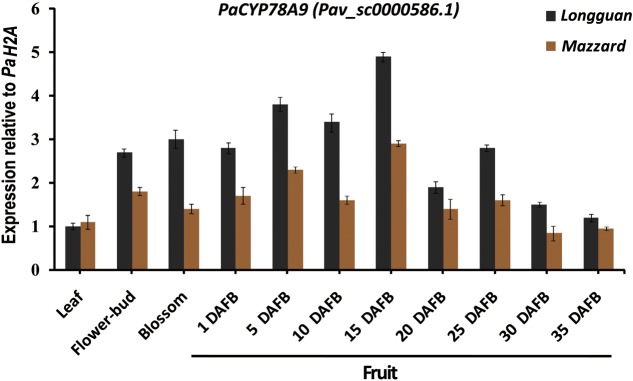
Expression profile of *PaCYP78A9* in the ‘Longguan’ landrace sweet cherry and ‘Mazzard’ wild sweet cherry cultivars. The expression levels of *PaCYP78A9* in leaves, flower-buds, blossoms, and fruit harvested at 1, 5, 10, 15, 20, and 25 days after full bloom (DAFB) were analyzed using qRT-PCR. Expression of *PaCYP78A9* in the mature leaves of ‘Longguan’ was set to 1.0. *P. avium Histone2A* (Pav_sc0000671.1) was used as an internal control to calculate the relative expression levels of *PaCYP78A9*. The error bars represent the standard deviation of three independent experiments.

To analyze the function of PaCYP78A9 during fruit growth and development, qRT-PCR was used to examine the expression pattern of *PaCYP78A9* in leaf, flower-bud, blossom, and fruit tissue obtained at different growth and developmental stages from both the wild sweet cherry ‘Mazzard’ and the landrace sweet cherry ‘Longguan.’ The expression level of *PaCYP78A9* was low in the leaves of both ‘Mazzard’ and ‘Longguan’ but was relatively high in the flower-bud and flowers of both lines during the blossom stages (**Figure 2**). During early fruit growth and development (1–25 DAFB) of ‘Mazzard’ and ‘Longguan,’ *PaCYP78A9* expression was more highly upregulated in fruit than in leaves, with expression peaking in fruit at 15 DAFB, suggesting that *PaCYP78A9* plays an important role in fruit growth and development (**Figure 2**). Furthermore, the fruit transcript levels of *PaCYP78A9* in the landrace sweet cherry ‘Longguan’ were significantly higher than in the wild sweet cherry ‘Mazzard’ (**Figure 2**). In *Arabidopsis*, *AtCYP78A9* was expressed only in floral organs, not in vegetative tissue (Sotelo-Silveira et al., 2013). Similarly, the *PaCYP78A9* gene was specifically expressed in sweet cherry floral organs that were related to the developing fruit. Together, these results indicate that the specific expression pattern of *PaCYP78A9* could be involved in regulating fruit expansion and ultimately fruit size during the early stages of fruit growth and development.

### Silencing of *PaCYP78A9* Decreases Fruit Size in Sweet Cherry

**FIGURE 3:**
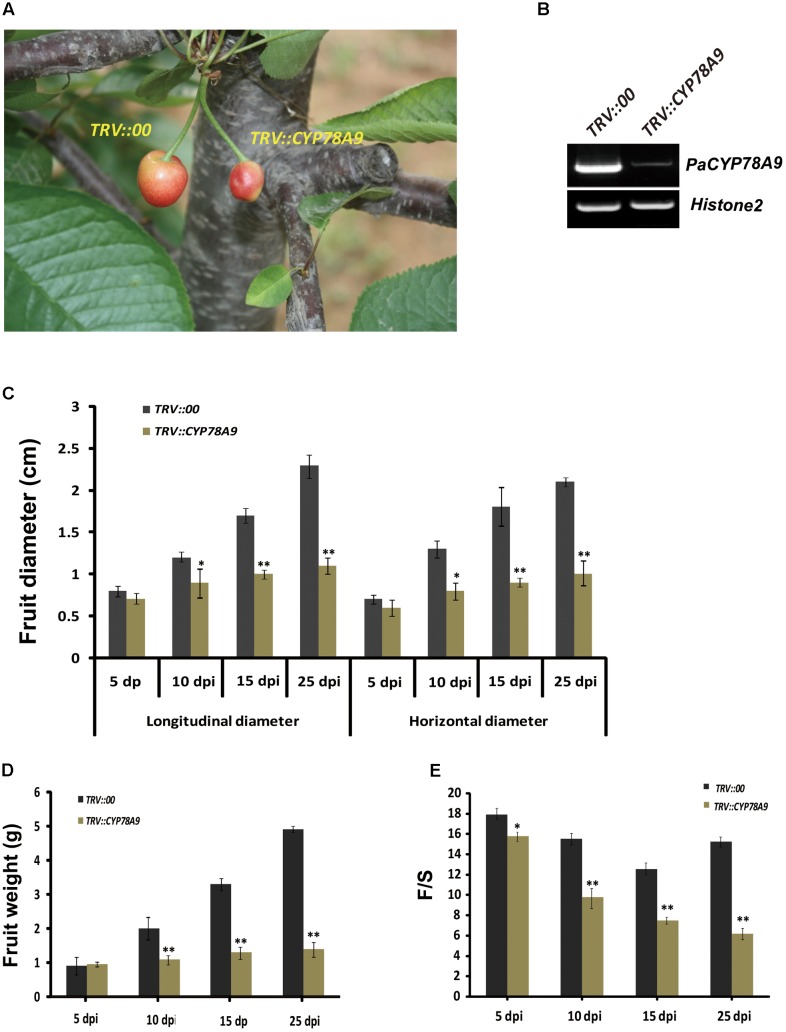
Silencing of *PaCYP78A9* decreased fruit size in sweet cherry. **(A)** The phenotype of fruit infected with *TRV::PaCYP78A9* or *TRV::00* at 25 days post-inoculation (dpi). **(B)** Pa*CYP78A9* transcript levels in the fruit of sweet cherry *TRV::PaCYP78A9*- and *TRV::00*-silencing lines at 15 dpi by semi-quantitative RT-PCR. *Histone2A* was used as an internal control. **(C–E)** The fruit longitudinal diameter **(C)**, horizontal diameter **(D)**, and the weight ratio of fruit flesh and fruit stone (F/S, **E**) of the *TRV::PaCYP78A9*-infected fruit were analyzed at 5, 10, 15, and 25 dpi. Values represent the mean ± SD from three independent replicates. Statistical significance was determined by the Student’s *t*-test. ^∗∗^*P*-value < 0.01, ^∗^*P*-value < 0.05.

Previous studies have confirmed that *Arabidopsis AtCYP78A9* control seed size by promoting cell proliferation (Sotelo-Silveira et al., 2013). To determine whether *PaCYP78A9* influences fruit size during fruit growth and development, the *tobacco rattle virus*-induced gene silencing (TRV-VIGS) technique was used to knock down expression of the *PaCYP78A9* gene in the sweet cherry landrace ‘Longguan.’. RT-PCR was performed using cDNA samples from infiltrated fruit at 15 dpi to verify that the *PaCYP78A9* gene in ‘Longguan’ was effectively silenced. This showed that the expression of *PaCYP78A9* was markedly reduced in the *TRV::PaCYP78A9*-infected fruit compared with the *TRV::00*-infected fruit (**Figure 3B**).

Investigation of plant phenotypes, measurement, and statistical analysis showed that the *TRV::PaCYP78A9*-infected fruit were dramatically smaller than the *TRV::00-*infected fruit at 5, 10, 15, and 25 dpi (**Figure 3**). The longitudinal diameter of the *TRV::PaCYP78A9*-infected fruit was reduced by 12, 38, 50, and 60%, respectively, when compared with the *TRV::00*-infected fruit at 5, 10, 15, and 25 dpi. Similarly, the horizontal diameter of *TRV::PaCYP78A9*-infected fruit also observably reduced (**Figure 3C**). Furthermore, the weight ratio of fruit flesh and fruit stone (F/S) of the *TRV::PaCYP78A9*-infected fruit were significantly reduced compared with the *TRV::00*-infected fruit (**Figures 3D,E**). However, there was no distinct change in the fruit stone size between *TRV::PaCYP78A9-*infected and *TRV::00*-infected sweet cherry fruits. These results suggest that the *PaCYP78A9* gene is a significant positive regulator of sweet cherry fruit size during the stages of fruit growth and development.

### Mesocarp Cell Number Is Reduced in *PaCYP78A9*-Silenced Sweet Cherry Fruit

**FIGURE 4:**
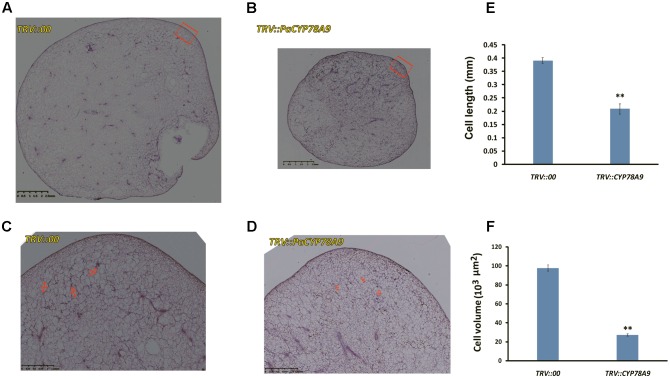
The tissue morphology of flesh taken from mesocarp longitudinal sections of sweet cherry fruit. **(A,B)** Mesocarp longitudinal sections from the fresh fruit of *TRV::00*-silencing **(A)** and *TRV::PaCYP78A9*-silencing **(B)** lines at 25 dpi stained with hematoxylin (Scale bars = 2.5 mm). **(C,D)** Magnified views of the mesocarp longitudinal sections (the red virtual box area) corresponding to **(A,B)** (Scale bars = 1.25 mm). **(E,F)** Quantification of cell length and Cell volume of the sections from the fresh fruit of *TRV::00*-silencing and *TRV::PaCYP78A9*-silencing lines at 25 dpi. Values represent the mean ± SD from three independent replicates. ^∗∗^Significant differences as calculated using the Student’s *t*-test at *p* < 0.01.

**FIGURE 5:**
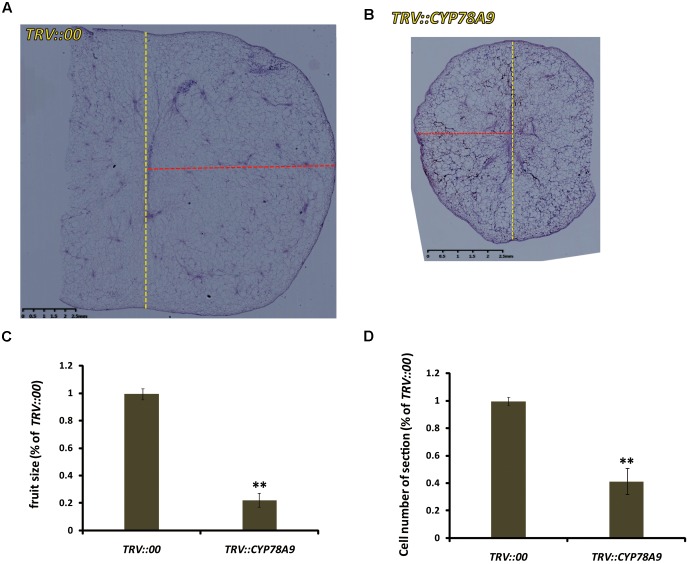
The tissue morphology of mesocarp equatorial sections taken from fresh sweet cherry fresh fruit. **(A,B)** Mesocarp equatorial sections from the fruit of *TRV::00*-silencing **(A)** and *TRV::PaCYP78A9*-silencing **(B)** lines at 25 dpi stained with hematoxylin (Scale bars = 2.5 mm). The yellow dashed lines showed the meridian of the fruit, the red dashed lines showed the half of equator of the fruit. **(C,D)** Quantification of the fruit size **(C)** and cell number of the sections **(D)** from the fresh fruit of *TRV::PaCYP78A9*-silencing lines compared with those *TRV::00*-silencing lines at 25 dpi. ^∗∗^Indicate significant differences from *TRV::00*-silencing fruit at *P* < 0.01 (*t*-test).

Some *CYP78A* family members, such as *TaCYP78A3*, *TaCYP78A5*, *AtCYP78A9*, and *AtCYP78A5*, influenced ultimate seed size by regulating cell proliferation in the integument, which is the future seed coat, in *Arabidopsis* and wheat (Adamski et al., 2009; Sotelo-Silveira et al., 2013; Ma et al., 2015, 2016). To determine whether the decreased fruit size observed in sweet cherry *PaCYP78A9*-silencing lines was caused by reduced cell volume in the ovary wall, the mesocarp cell volume of fruit from the *TRV::PaCYP78A9-* and *TRV::00*-silencing sweet cherry lines was examined. Mesocarp cell volumes were significantly reduced in fruit from sweet cherry *PaCYP78A9*-silencing lines compared with fruit from the *TRV::00*-silencing lines at 25 dpi (**Figures 4**, **5**). Furthermore, the mesocarp cell length and fruit size were smaller for the *PaCYP78A9*-silencing lines than the *TRV::00*-silencing lines (**Figures 4C,D**). Therefore, the decreased fruit size could be caused by a reduction in the mesocarp cell volume and expansion.

### Downregulation of Cell Cycle Genes in Sweet Cherry Fruit from *PaCYP78A9*-Silencing Lines

**FIGURE 6:**
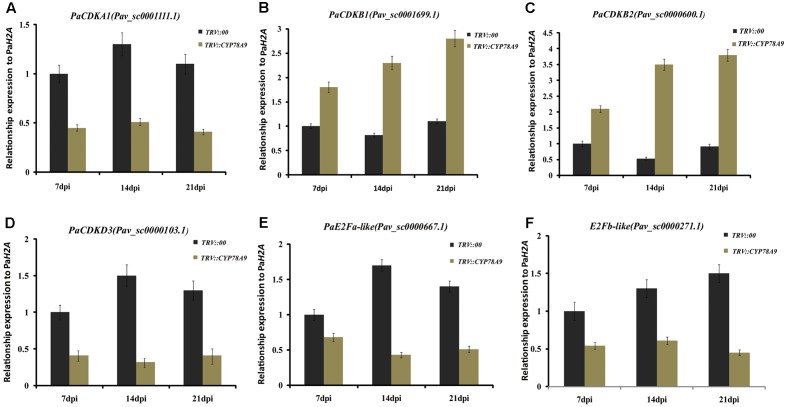
Quantitative real-time PCR analysis showing the expression patterns of cyclin-dependent kinase (*CDKA1*, *CDKB1*, *CDKB2*, and *CDKD3*) and transcription factors encoding promoters of the cell cycle (*E2Fa-like* and *E2Fb-like*) in fruit from the sweet cherry *TRV::PaCYP78A9*- and *TRV::00*-silencing lines at 7, 14, and 21 dpi. The expression of *CDKA1* **(A)**, *CDKD3* **(D)**, E2Fa-like **(E)**, and E2Fb-like **(F)** in *TRV::PaCYP78A9*-silenced fruit at 7, 14, and 21 dpi was downregulated compared with their expression in the fruit of *TRV::00*-silencing lines. Conversely, the transcript levels of *CDKB1* **(B)** and *CDKB2* **(C)** increased in *TRV::PaCYP78A9*-silenced fruit compared with *TRV::00*-silenced fruit. The gene expression in *TRV::00*-silenced sweet cherry fruit at 7 dpi was set to 1.0 and *Histone2A* (Pav_sc0000671.1) was used as an internal standard. Values are means ± SD of three independent experiments performed in duplicate.

**FIGURE 7:**
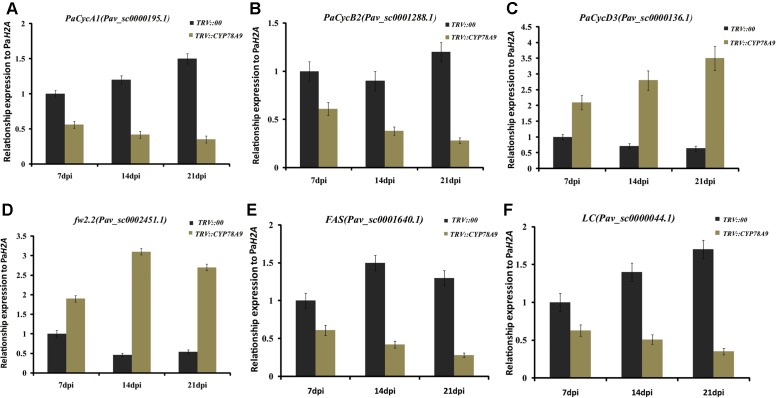
Quantitative real-time PCR analysis of the expression levels of cyclins (*CycA1, CycB2*, and *CycD3*) and other cell division regulation factors (*fw2.2*, *FAS*, and *LC*) in the fruit for sweet cherry *TRV::PaCYP78A9*- and *TRV::00*-silencing lines at 7, 14, and 21 dpi. The expression of *CycA1* **(A)**, *CycB2* **(B)**, *FAS* **(E)**, and *LC* **(F)** at 7, 14, and 21 dpi in *TRV::PaCYP78A9*-silenced fruit was downregulated when compared with their expression in fruit of the *TRV::00*-silencing lines. Conversely, *CycD3* **(C)** and *fw2.2* **(D)** were upregulated in *TRV::PaCYP78A9*-silenced fruit compared with *TRV::00*-silenced fruit. Gene expression in *TRV::00*-silenced sweet cherry fruit at 7 dpi was set to 1.0 and *Histone2A* (Pav_sc0000671.1) was used as an internal standard. Values are means ± SD of three independent experiments performed in duplicate.

Previous studies have shown that cell division activity was restricted by decreases in the transcript levels of cell proliferation-related marker genes and cell cycle genes, including cyclin-dependent kinases (*CDKA1*, *CDKB1*, *CDKB2*, and *CDKD3*), cyclins (*CycA1*, *CycB2, and CycD3*), transcription factors (*E2Fa-like* and *E2Fb-like*) encoding promoters of the cell cycle, and other cell division regulation factors such as the cell number regulator 1-like factor (*fw2.2*), *FASCIATED* (*FAS*), and LOCULE NUMBER (*LC*); downregulation of these genes ultimately resulted in small fruit (Inzé and De Veylder, 2006; Rodríguez et al., 2011; Azzi et al., 2015; Okello et al., 2015). To further analyze the function of *PaCYP78A9*, the transcript levels of the cell cycle genes and cell proliferation-related marker genes were detected in the fruit of sweet cherry *TRV::PaCYP78A9*- and *TRV::00*-silencing lines using qRT-PCR. Notably, in *TRV::PaCYP78A9*-silencing fruit, the expression level of *CDKA1* (Pav_sc0001111.1), *CDKD3* (Pav_sc0000103.1), *CycA1* (Pav_sc0000195.1), *CycB2* (Pav_sc0001288.1), *E2Fa-like* (Pav_sc0000667.1), *E2Fb-like* (Pav_sc0000271.1), *FAS* (Pav_sc0001640.1), and *LC* (Pav_sc0000044.1) were significantly lower than in the fruit of *TRV::00*-silencing lines (**Figures 6**, **7**). Conversely, the transcript levels of *CDKB1* (Pav_sc0001699.1), *CDKB2* (Pav_sc0000600.1), *CycD3* (Pav_sc0000136.1), and *fw2.2* (Pav_sc0002451.1) were higher in the fruit of *TRV::PaCYP78A9*-silencing lines compared with the fruit of *TRV::00*-silencing lines (**Figures 6**, **7**). Previous studies have shown that overexpression of *CycD3* reduced cell proliferation in *Arabidopsis* and that *FW2.2* and *CDKB1*/*2* negatively regulated fruit size by controlling cell proliferation during early fruit development in tomato (Frary et al., 2000; Czerednik et al., 2012). Similarly, these genes appear to show analogous functions in the regulation of fruit size in cherry. The downregulation of most cell cycle and proliferation genes in the fruit of *TRV::PaCYP78A9*-silencing lines suggests that *PaCYP78A9* is likely to an important upstream protein factor of cell cycle processes.

### Overexpression of *PaCYP78A9* Leads to Increased Seed Size in *Arabidopsis*

In *Arabidopsis*, overexpression of *CYP78A9* previously resulted in large and seedless siliques and in larger seeds. Moreover, *CYP78A9* was highly expressed in the floral organs, especially in the inner carpel wall, suggesting that *CYP78A9* plays a role in fruit development (Sotelo-Silveira et al., 2013). To further characterize the function of *PaCYP78A9* in this study, the overexpression vector *p35S::PaCYP78A9* was assembled and introduced into wild-type (WT) Columbia *Arabidopsis* plants via *Agrobacterium*-mediated transformation. We observed that 20 T2 transgenic lines had larger siliques and seeds when compared with WT Columbia *Arabidopsis*; this increase in silique size was positively correlated with the expression level of *PaCYP78A9* in the different transgenic lines (**Supplementary Figure S1**). Three independent T3 transgenic lines with the highest constitutive *PaCYP78A9* expression, *PaCYP78A9-3*, *PaCYP78A9-5*, and *PaCYP78A9-11* were selected for further analysis.

**FIGURE 8:**
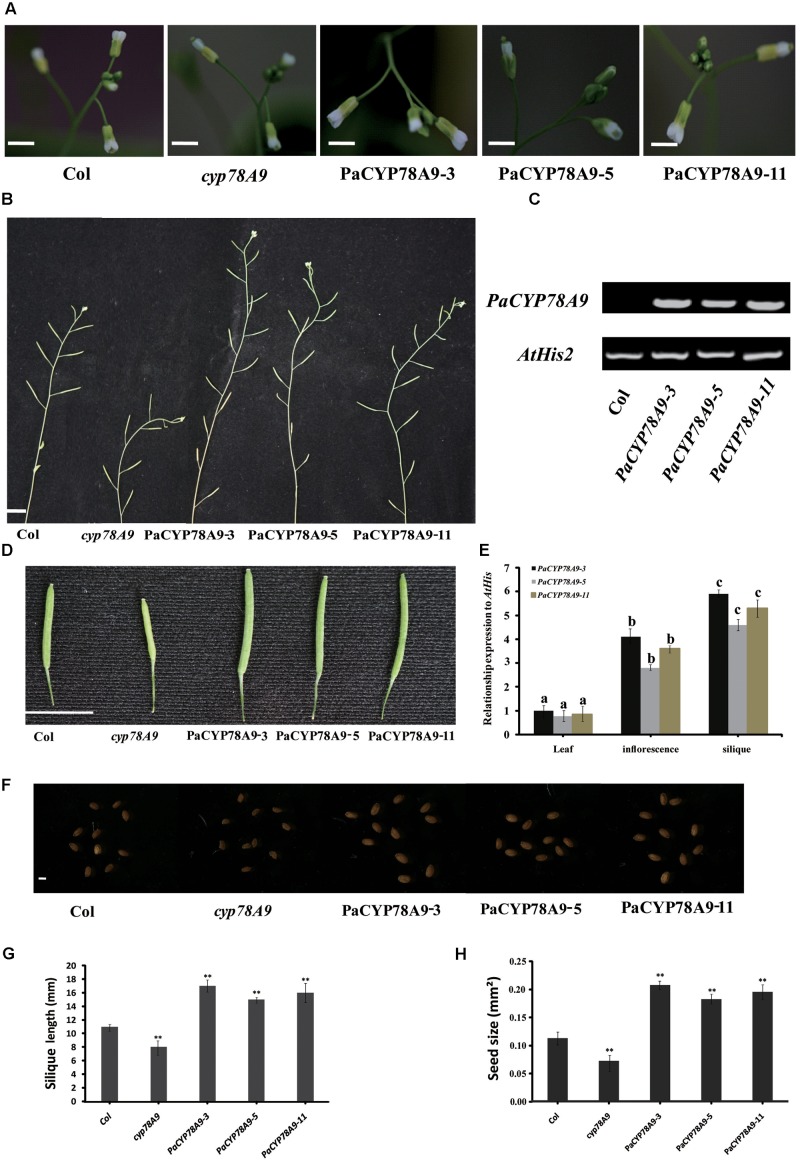
Overexpression *PaCYP78A9* increased silique and seed size in *Arabidopsis.* **(A)** Inflorescence phenotypes of transgenic *PaCYP78A9* overexpression lines, *cyp78A9* mutant lines, and wild-type (WT) *Arabidopsis* (scale bars = 2.00 mm). **(B)** The phenotypes of the main inflorescence stem at stage 6.00 (The stage is in accordance with those described by Boyes et al., 2001) in *PaCYP78A9* overexpression lines, *cyp78A9* mutant lines, and WT (scale bar = 10.00 mm). **(C)** RT-PCR analysis of *PaCYP78A9* in *Arabidopsis* transgenic lines. Total RNA was isolated from a mixture of inflorescences and siliques from 10 plants per line. **(D)** Silique phenotypes (scale bar = 10.00 mm). **(E)** Expression levels of *PaCYP78A9* in different tissues from T3 transgenic lines. **(F)** Seed phenotypes (scale bar = 0.1 mm). **(G,H)** All siliques **(G)** and seeds **(H)** were measured for 10 transgenic *Arabidopsis* plants, *cyp78A9* mutants, and WT plants. **(H)** Seed size as represented by the projected area of a seed. Values represent the mean ± SD from three independent replicates. Statistical significance **(G,H)** was determined by the Student’s *t*-test, ^∗∗^significant differences as calculated using the Student’s *t*-test at *p* < 0.01; and **(E)** by one-way ANOVA, letters indicate significant differences (*P* < 0.05, Duncan test).

While none of the transgenic overexpression lines displayed morphological or growth differences in the vegetative stage when compared with WT *Arabidopsis* plants, the flowers of these overexpression lines were larger, and they produced more siliques than the WT plants (**Figures 8A,B,D,G**). The seeds of all T3 *PaCYP78A9* overexpression transgenic lines were significantly larger than those of WT plants, with both increased seed widths and lengths (**Figures 8F,H**). The shape of these seeds did not, however, observably change when compared with seed from the WT plants.

We also examined the expression patterns of *PaCYP78A9* in the developing flowers and seeds of three independent transgenic T3 overexpression lines and the WT control. Using different tissues from these transgenic lines, qRT-PCR indicated that *PaCYP78A9* was largely detected in the inflorescences and siliques of transgenic T3 plants (**Figure 8E**). Moreover, seed size was correlated with the expression level of *PaCYP78A9* in the different transgenic lines (**Supplementary Figure S1** and **Figure 8**). These results indicate that overexpression of *PaCYP78A9* promoted large seeds; combined with evidence that silencing of *PaCYP78A9* decreased cherry fruit size, this suggests that *PaCYP78A9* plays an essential role in the growth and development of reproductive organs in sweet cherry.

### *PaCYP78A9* Can Rescue the Phenotype of the *Arabidopsis Cyp78a9* Deletion Mutant

**FIGURE 9:**
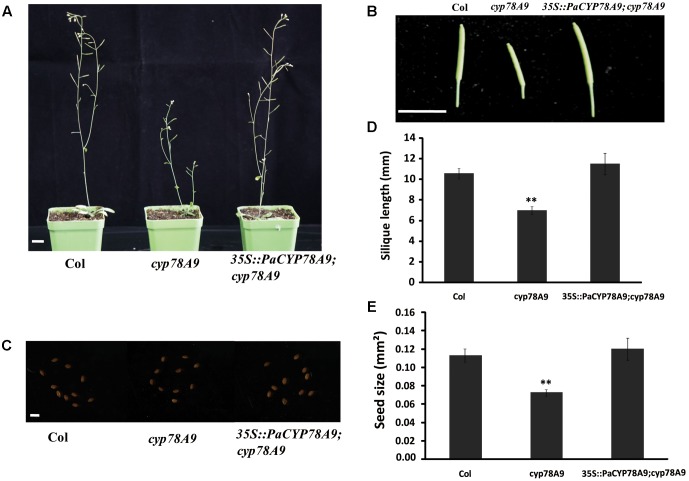
*PaCYP78A9* can rescue the phenotype of the *Arabidopsis cyp78a9* deletion mutant. **(A)** The phenotype of *Arabidopsis* plants at stage 6.00 (scale bar = 10.00 mm). **(B)** Silique phenotypes (scale bar = 10.00 mm). **(C)** Seed phenotypes (scale bar = 0.1 mm). **(D,E)** All siliques **(D)** and seeds **(E)** were measured for 10 plants of each genotype. Values represent the mean ± SD from three independent replicates. ^∗∗^Significant differences as calculated using the Student’s *t*-test at *p* < 0.01.

To further demonstrate the function of *PaCYP78A9* in promoting fruit growth and expansion in sweet cherry, a functional copy of *PaCYP78A9* containing the promoter *CaMV 35S* and terminator *NOS* was transformed into homozygous *cyp78a9* mutants (Salk_066588C, obtained from the ABRC) in the Columbia-0 background that display a small seed phenotype. The seed size of *cyp78a9* mutants could be rescued to normal WT *Arabidopsis* levels through the expression of *PaCYP78A9* in these lines (**Figure 9**). Furthermore, overexpression of *PaCYP78A9* in the *cyp78a9* mutant resulted in seeds that were larger than those of WT *Arabidopsis* (**Figure 9C**). These results indicate that *PaCYP78A9* can rescue the defective phenotype of the *Arabidopsis cyp78a9* insertional mutant, suggesting a conserved function for these two genes in promoting organ size.

## Discussion

Cytochrome P450 is one of the largest families of plant proteins; the involvement of its members in the regulation of various metabolic pathways affecting plant growth and development has been studied systematically in many organisms (Ohkawa et al., 1998; Xu J. et al., 2015). Seven plant CYP78A family members, such as CYP78A5, CYP78A6, CYP78A9, CYP78A11, CYP78A13, and CYP78A3 have been identified in *Arabidopsis*, rice, wheat, tomato, *Zea mays*, and *Physcomitrella patents* where they were found to be involved in promoting and enhancing growth and development of the female reproductive organs (Imaishi et al., 2000; Adamski et al., 2009; Katsumata et al., 2011; Fang et al., 2012; Chakrabarti et al., 2013; Nagasawa et al., 2013; Sotelo-Silveira et al., 2013; Ma et al., 2015, 2016; Xu F. et al., 2015). However, the role of CYP78A members in controlling organ size and development in fruit trees was less well characterized.

In this study, we characterized the biological function of the *P. avium PaCYP78A9* gene. Overexpression of *PaCYP78A9* in *Arabidopsis* resulted in increased seed size, with the *PaCYP78A9* gene found to be largely expressed in the inflorescences and siliques of transgenic plants, and in the flowers and fruit of sweet cherry. Sweet cherry lines silenced for *PaCYP78A9* using RNAi produced smaller fruit than the control plants. Moreover, PaCYP78A9 influenced fruit size by affecting mesocarp cell proliferation and expansion during fruit growth and development. Our findings indicate that PaCYP78A9 plays an essential role in regulating cherry fruit size, thereby providing insights into the molecular basis underlying fruit traits such as fruit size and other horticultural characteristics in *P. avium*.

Although the development of seeds is a complex process, recent studies have shown that final seed size is influenced primarily by three major programs; the bi-parentally derived fertilization products, namely the embryo and endosperm, and the seed coat that is derived from the ovule integument in *Arabidopsis*, wheat, and rice (Ohto et al., 2005; Sundaresan, 2005; Berger et al., 2006). Some CYP78As and transcription factors such as DA1, TTG2, ARF2, AP2, AtCYP78A9/TaCYP78A3, EOD3/CYP78A6, CYP78A5/KLU, and a KLU homolog affected seed size by promoting cell proliferation of the ovule integuments (seed coat) (Adamski et al., 2009; Sotelo-Silveira et al., 2013). Likewise, mature stone fruit (e.g., peach, cherry, and apricot) derived from ovary tissues are composed of pericarp (exocarp, mesocarp, and endocarp) and seed. Cell expansion and division are important factors regulating fruit size (Gillaspy et al., 1993). In cherry, previous research suggested that fruit size within the same genotype was controlled through mesocarp cell size (Olmstead et al., 2007; Zhang and Whiting, 2011). Conversely, our results indicate that final fruit size was regulated by accelerating mesocarp cell proliferation and expansion during fruit growth and development. Interestingly, overexpression of PaCYP78A9 in *Arabidopsis* resulted in both enlarged siliques and seeds but also lengthened the reproduction phase. In *Arabidopsis*, CYP78A9 is involved in controlling floral organ size and also functions during reproductive development by participating in the conversion of dihydrokaempferol to dihydroquercetin in the flavonoid pathway (Sotelo-Silveira et al., 2013). In view of the above findings, we speculate that PaCYP78A9 maybe function by producing a signal of some sort, or by participating in the pathway regulating reproductive organ size through the proliferation of mesocarp cells during fruit growth and development. Taken together, our findings indicate that PaCYP78A9, like CYP78A family members in other plants, regulated reproductive organ development.

While we have characterization the function of CYP78A9 in the regulation of organ size, the regulation mechanism and genetic network mediating CYP78A9 remains unclear. Moreover, CYP78A subfamily genes are generally expressed in floral organs or specific meristems where they move within an inflorescence, not between inflorescences (Zondlo and Irish, 1999; Eriksson et al., 2010; Rolland-Lagan, 2010). For example, *CYP78A9* expression in *Arabidopsis* was detected only in floral organs and not in vegetative tissue (Sotelo-Silveira et al., 2013). Furthermore, CYP78A5/KLU positively regulated a mobile signal downstream from KLU within an inflorescence that affected flower size by influencing cell proliferation (Rolland-Lagan, 2010). To uncover the genetic regulatory network of CYP78A9, the transcript level of cyclin-dependent kinases, cyclins, transcription factors that encode promoters of the cell cycle, and other cell division regulation factors involved in cell proliferation and the regulation of cell cycle genes were analyzed. Our results showed that, with the exclusion of *CDKB1*, *CDKB2*, *CycD3*, and *fw2.2*, cell cycle genes and transcription factors were most downregulated when *PaCYP78A9* was silenced in sweet cherry fruit. During organ growth and development, plant cell proliferation was regulated by the cell cycle; this endoreduplication is largely controlled by Cyc and CDK dimers while cell expansion is related to endoreduplication (Joubes and Chevalier, 2000; Sugimoto-Shirasu and Roberts, 2003; Renaudin et al., 2017). Moreover, the size and number of fruit pericarp cells that were associated with endoreduplication in tomato influenced final tomato fruit size (Cheniclet et al., 2005; Gonzalez et al., 2007; Su et al., 2014; Musseau et al., 2017). Knock-down of CDKA1 and overexpression of either CDKB1 or CDKB2 resulted in smaller and irregularly shaped fruits by reducing the number of exocarp cell layers (Czerednik et al., 2012, 2015). Overexpression of cyclins *CycA1, CycB2*, and *CycD3;3* enhanced growth rate by promoting cell proliferation (Doerner et al., 1996; Dewitte et al., 2007), and the transcription factors E2Fa and E2Fb, acting as activators of cell proliferation, played an important role in cell cycle progression and development (Sozzani et al., 2006). Furthermore, both FAS and LC were ultimately responsible for the growth of larger fruit by increasing floral meristem size and cell division (Cong et al., 2008; Muños et al., 2011). In this study, the expression of positive cell proliferation and cell expansion regulators, including two cyclin-dependent kinases (*CDKA1* and *CDKD3*), three cyclins (*CycA1, CycB2*, and *CycD3;3*), two transcription factors (*E2Fa* and *E2Fb*) and two other cell division regulation factors (*FAS* and *LC*) was suppressed in cherry fruit with reduced *PaCYP78A9* expression. We, therefore, hypothesize that *PaCYP78A9* is an upstream regulator of during fruit growth and development. Previous studies have indicated that many CYPs are involved in the metabolism of different phytohormones and that CYP78A might be a novel mobile factor regulating organ growth and development in a manner similar to these phytohormones (Adamski et al., 2009; Xu J. et al., 2015). However, the mechanisms underlying the *PaCYP78A9*-mediated regulation of cell proliferation and cell expansion require further investigation, with more efforts needed in the future to characterize the mechanism of *CYP78A9*.

In summary, PaCYP78A9, an ortholog of *Arabidopsis* CYP78A9 in sweet cherry, was functionally characterized and found to affect fruit size by influencing mesocarp cell proliferation and expansion during fruit growth and development. The specific transcription pattern of *PaCYP78A9* was positively and closely associated with final fruit size and we further used transgenic lines over-expressing or silenced for *PaCYP78A9* to show that it is involved in regulating fruit size. These findings provide novel insights into understanding the molecular mechanisms underlying fruit size determination during fruit growth and development in fruit trees.

## Author Contributions

ML, XQ, and CL conceived the research. XQ designed the experiments. XQ, CL, LS, and YL performed the experiments. XQ analyzed the results, and wrote the manuscript. ML provided scientific suggestions, and revised the manuscript. All authors approved the final manuscript, and agreed to be accountable for all aspects of the work in ensuring that questions related to the accuracy or integrity of any part of the work are appropriately investigated and resolved.

## Conflict of Interest Statement

The authors declare that the research was conducted in the absence of any commercial or financial relationships that could be construed as a potential conflict of interest.

## Footnotes

1 http://www.rosaceae.org/gb/gbrowse/prunus_persica_v2.1

## Supplementary Material

FIGURE S1 Quantitative real-time PCR (qRT-PCR) analysis of the expression level **(A)** of *PaCYP78A9* and seed size **(B)** of PaCYP78A9 over-expression T2 *Arabidopsis* transgenic lines compared with wild *Arabidopsis*. The endogenous *ATCYP78A9* expression in WT *Arabidopsis* was set to 1.0. (*t*-test: ^∗^*P* < 0.05, ^∗∗^*P* < 0.01, ^∗∗∗^*P* < 0.001).
