# Supplementary figures and images for: PaCYP78A9, a Cytochrome P450, Regulates Fruit Size in Sweet Cherry (Prunus avium L.)
Source: Front Plant Sci. 2017 Dec 5;8:2076. doi: 10.3389/fpls.2017.02076 (PMC5723407; doi:10.3389/fpls.2017.02076)

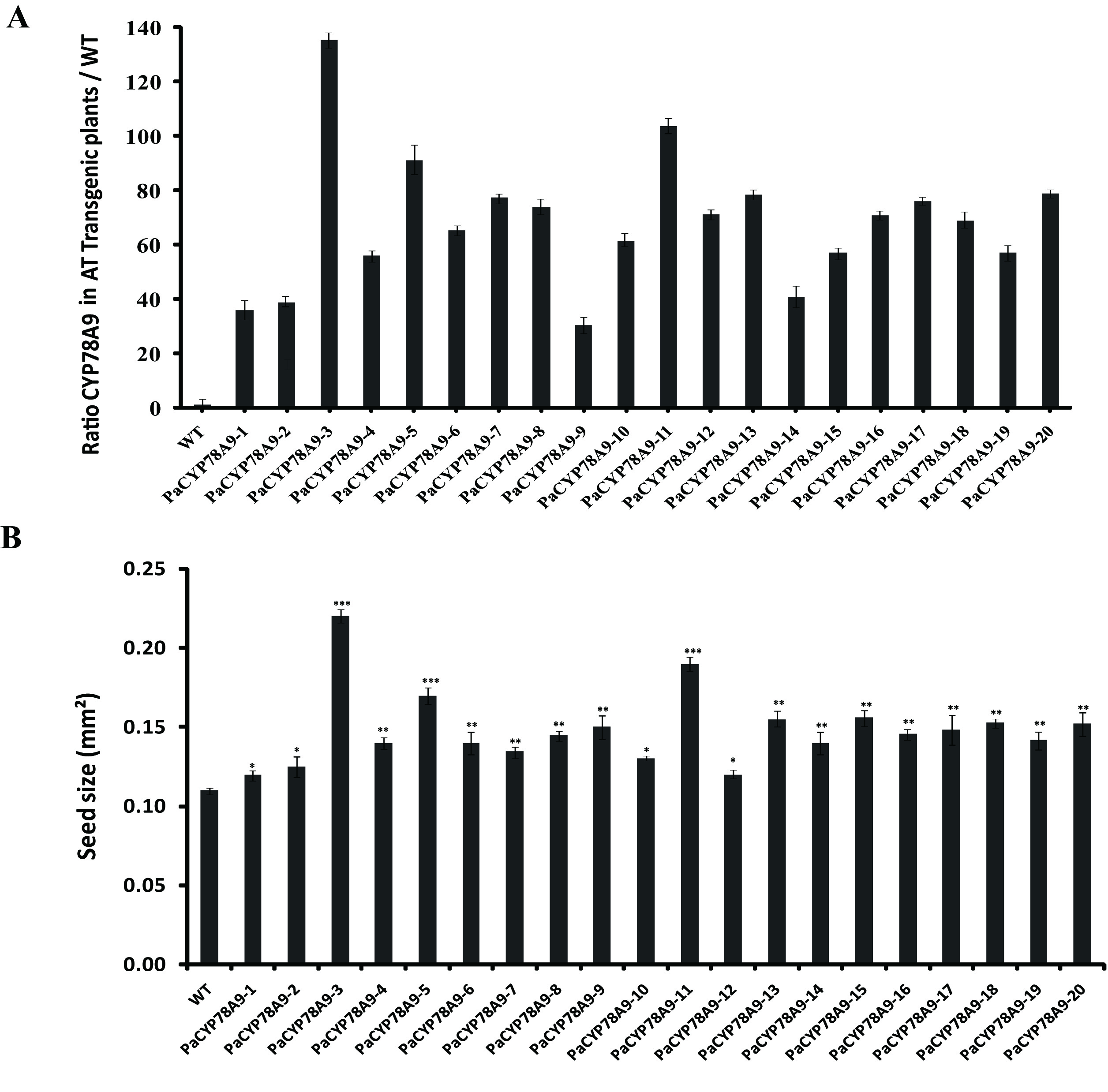

Supplement: FIGURE S1 — Quantitative real-time PCR (qRT-PCR) analysis of the expression level (A) of PaCYP78A9 and seed size (B) of PaCYP78A9 over-expression T2 Arabidopsis transgenic lines compared with wild Arabidopsis. The endogenous ATCYP78A9 expression in WT Arabidopsis was set to 1.0. (t-test: ∗P < 0.05, ∗∗P < 0.01, ∗∗∗P < 0.001). [file Image_1.JPEG]
