# Supplementary material for: PaCYP78A9, a Cytochrome P450, Regulates Fruit Size in Sweet Cherry (Prunus avium L.)
Source: Front Plant Sci. 2017 Dec 5;8:2076. doi: 10.3389/fpls.2017.02076 (PMC5723407; doi:10.3389/fpls.2017.02076)
Supplement: Supplementary file 2 [file Table_1.DOCX]

**Table1**: Primers used in this study.

| Primer name | Sequences (5ʹ→3ʹ) |
| --- | --- |
| PaCYP78A9-F | CTCTCCGCCATGAAAACAGAC |
| PaCYP78A9-R | CGGGCGCACTTTAGCAACC |
| PaCYP78A9-e-F | GAACACGGGGGACTCTAGACTCTCCGCCATGAAAACAGAC |
| PaCYP78A9-e-R | CTGACCACCCGGGGATCC CGGGCGCACTTTAGCAACC |
| PaCYP78A9-RNAi-F | GGAATTCCCGCCGCCCATCTCTTCTC |
| PaCYP78A9-RNAi-R | GGGTACCTCGGACAATTTATCGGGACCTT |
| Histone2-F | GGTGTGCTTCCGCAGATAA |
| Histone2-R | TCCTCCTTGGGTGGTGAAT |
| CDKA1-F | GTTGGTTTACAAGGCTCGTGA |
| CDKA1-R | CAGCCTGACAATGTTGCCA |
| CDKB1-F | TCTCAAGAGTTACACGCACGA |
| CDKB1-R | TGCTTGTCTGTTGGTGTTCCT |
| CDKB2-F | TACAACAACAGTGTCGGCG |
| CDKB2-R | CCCTTGTTTCACATCCATCAAC |
| CDKD3-F | ATTTGCTGAACTTCTCCTACGC |
| CDKD3-R | CATCTTCGCTAACAAATCCAGG |
| CycA1-F | ATTACACGCTTTATCAGCCGTC |
| CycA1-R | CCACTTTCTTACGGTCAGCACTA |
| CycB2-F | GCCGATAATGAGTTGGCAGTA |
| CycB2-R | GCTGATGCCAACTAACTGAAGC |
| CycD3-F | AAACGAAGCACCAGAGCAC |
| CycD3-R | CCAAGGAGAGACAGGTGACA |
| E2Fa-like -F | CGAAAGTGGCTGTTTGTCAC |
| E2Fa-like -R | CCACTTCTGTTGTTGGGTGT |
| E2Fb-like - F | AGAAGCACGATGGGTCCAA |
| E2Fb-like - R | CCCTCTATTCTCTGTGACCATTG |
| fw2.2-F | TGGTCTTTGCCATTGTTGTG |
| fw2.2-R | CGGTAGAAGCACGAATACAAGC |
| FAS-F | CGGGTCTTCATCATCTTCCA |
| FAS-R | GAACTCGTTGTCTCTTCTCGG |
| LC-F | GGCAAGATTGAAGGCAAGAAC |
| LC-R | TGCCCAACAGCAATCACA |
| AtHis2-F | TCAGTTACGCTTCATCCTCC |
| AtHis2-R | CGGCGAGGTATTCAAGAAC |

Note: The NCBI references of the genes in supplemental table 1 were as follows: *PaCYP78A9* ([XM_021959332.1](https://www.ncbi.nlm.nih.gov/nucleotide/1220057722?report=genbank&log$=nuclalign&blast_rank=1&RID=Y8M95PSS014)), *Histone2* (Pav_sc0000671.1), *CDKA1* (Pav_sc0001111.1) *CDKB1* (Pav_sc0001699.1), *CDKB2* (Pav_sc0000600.1), *CDKD3* (Pav_sc0000103.1), *CycA1* (Pav_sc0000195.1), *CycB2* (Pav_sc0001288.1), *CycD3* (Pav_sc0000136.1), *E2Fa-like* (Pav_sc0000667.1), *E2Fb-like* (Pav_sc0000271.1), *fw2.2* (Pav_sc0002451.1), *FAS* (Pav_sc0001640.1), *LC* (Pav_sc0000044.1), *AtHis2* (NM_118857.4).
